# Role of the cGAS-STING Pathway in Aging-related Endothelial Dysfunction

**DOI:** 10.14336/AD.2022.0316

**Published:** 2022-12-01

**Authors:** Huilin Yu, Ke Liao, Yu Hu, Dingyi Lv, Minghao Luo, Qian Liu, Longxiang Huang, Suxin Luo

**Affiliations:** ^1^Department of Cardiology, The First Affiliated Hospital of Chongqing Medical University, Chongqing, China; ^2^Institute of Life Science, Chongqing Medical University, Chongqing, China

**Keywords:** aging, cGAS-STING pathway, endothelial nitric oxide synthase, endothelial dysfunction

## Abstract

Endothelial dysfunction develops gradually with age, and is the foundation of many age-related diseases in the elderly. The purpose of this study was to investigate the role of the cyclic GMP-AMP synthase (cGAS)-stimulator of interferon genes (STING) pathway in aging-related endothelial dysfunction. Endothelial functional parameters and biochemical indices of vascular function were examined in 2-, 6-, 12- and 24-month-old mice. Then, 6-month-old mice were administered RU.521, a specific inhibitor of cGAS, for 6 months, and endothelial functional parameters and biochemical indices of vascular function were re-examined. An *in vitro* model of cell senescence was established by treating human aortic endothelial cells (HAECs) with D-Galactose (D-GAL). Using inhibitors or siRNA interference, cGAS and STING were suppressed or silenced in senescent HAECs, and changes in the expression of eNOS, the senescence markers, p53, p21 and p16, components of the cGAS-STING pathway and Senescence-Associated β-galactosidase (SA-β-gal) staining were examined. Finally, cGAS, STING and p-IRF3 levels were measured in aorta tissue sections from eight patients. A decline in endothelial function, up-regulation of p53, p21 and p16 expression, and activation of the cGAS-STING pathway were observed in aging mice. Inhibition of cGAS was found to improve endothelial function and reverse the increased expression of aging markers. Our *in vitro* data demonstrated that D-GAL induced a decrease in eNOS expression and cell senescence, which could be partly reversed by cGAS inhibitor, STING inhibitor, siRNA-cGAS and siRNA-STING treatment. Higher expression levels of cGAS, STING and p-IRF3 were observed in aged human aortic intima tissue compared to young aortic intima tissue. Our study demonstrated that activation of the cGAS-STING pathway played a vital role in aging-related endothelial dysfunction. Thus, the cGAS-STING pathway may be a potential target for the prevention of cardiovascular diseases in the elderly.

Over the past few decades, aging-related cardiovascular diseases have become an increasing health threat due to the increase in aging populations worldwide [1]. For example, it is estimated that in China approximately 270 million people, most of which are elderly, suffer from hypertension [2, 3]. Furthermore, studies have shown that coronary heart disease (CHD) accounts for approximately one-third of all deaths in people older than 35 years, despite early and effective interventions, such as percutaneous coronary intervention (PCI) [4, 5]. Although aging is a part of life, it is also, unfortunately, one of the most important risk factors for cardiovascular diseases [6]. Thus, the development of preventative strategies is crucial to limit the occurrence and severity of aging-related cardiovascular diseases.

Endothelial dysfunction, a leading cause of abnormal vasodilation, is the basis of many cardiovascular diseases [7]. Improving endothelial function has been shown to be beneficial in the prevention and treatment of hypertension and CHD [8, 9]. However, to date, little is known about the effects of existing methods on the prevention of endothelial dysfunction. Therefore, exploring the mechanism of endothelial dysfunction may be beneficial in the development of more effective therapeutic targets. Endothelial nitric oxide synthase (eNOS) is a vital enzyme required for NO synthesis in endothelial cells and is the primary regulator of homeostasis and vascular tone [10]. A decline in eNOS expression often leads to increased vascular tension and decreased local blood perfusion, which are responsible for the development of many cardiovascular diseases [11].

Sterile inflammation has been shown to occur with aging [12]. This type of inflammatory response is due to immune dysfunction and is closely associated with aging-related organ dysfunction [13]. Evidence suggests that the increase in pro-inflammatory factors caused by sterile inflammation reduces vascular eNOS expression and NO production, leading to vasodilation dysfunction and ultimately aging-related cardiovascular diseases [14, 15]. However, the mechanisms involved in the regulation of age-related aseptic inflammation need further exploration.

The cyclic GMP-AMP synthase (cGAS)-stimulator of interferon genes (STING) pathway is a newly discovered component of the innate immune system [16]. The cGAS acts as a cytoplasmic DNA sensor, and its activation leads to activation of the downstream target STING, and subsequent phosphorylation of interferon regulatory factor 3 (IRF3) [17]. The main targets of IRF3 are inflammatory genes such as interferon-β (IFNβ), interferon induced protein with tetratricopeptide repeats 1 (Ifit1), Ifit2, Ifit3, and monocyte chemoattractant protein-1 (MCP-1) [18, 19]. Increased transcription of pro-inflammatory cytokines, such as tumor necrosis factor alpha (TNF-α), interleukin-1β (IL-1β) and IL-6 eventually lead to sterile inflammation [20]. Sterile inflammation is similar to inflammation that appears during infection and may be responsible for tissue injury [21].

Although activation of the cGAS-STING pathway has been shown to be involved in degeneration of the nervous system and cardiac dysfunction caused by aging [22, 23], the role of the cGAS-STING pathway in aging-related endothelial dysfunction remains unknown. Here, we examined the relationship between the cGAS-STING pathway and aging-associated endothelial dysfunction.

## MATERIALS AND METHODS

### Antibodies and chemicals

Primary antibodies against eNOS (Cat# 32027), p53 (Cat# 2524), STING (applies to Western blot and immumohisto-chemical staining, Cat# 13647) and IRF3 (Cat# 4302) were purchased from Cell Signaling Technology (MA, USA). The primary antibodies against p-IRF3 (Ser396) (Cat# AF2436) and p-STING (Ser366) (Cat# AF7416) were purchased from Affinity Biosciences (OH, USA). Primary antibody against CD31 (applies to immunofluorescence staining, AF20112) was purchased from AiFang biological (Hunan, China). Primary antibodies against cGAS (reacts with mouse, Cat# ab252416) and CD31 (applies to immumohisto-chemical staining, Cat# ab281583) were obtained from Abcam (Cambridge, UK). Primary antibodies against cGAS (reacts with human, Cat# sc-515777), p16 (Cat# sc-1661) and p21 (Cat# sc-6246) were purchased from Santa Cruz Biotechnology (TX, USA). Primary antibodies against STING (applies to Immunofluorescence Staining, 19851-1-AP) and β-actin (Cat# 20536-1-AP) were purchased from Proteintech Group (IL, USA). Secondary antibodies conjugated with horseradish peroxidase (HRP) (goat anti-rabbit, Cat# 31460; goat anti-mouse, Cat# 62-6520) were purchased from Thermo Fisher Scientific (MA, USA). RU.521 (Cat# HY-114180), H-151 (Cat# HY-112693) and D-GAL (Cat# HY-N0210) were obtained from MCE (NJ, USA). FITC-conjugated goat anti-rabbit secondary antibody (Cat# A0562), Senescence β-Galactosidase Staining kit (Cat# C0602) and NO Detection kit (Cat# S0021S) were purchased from Beyotime (Shanghai, China).

### Animals

Healthy male C57BL/6 mice aged 2, 6, 12, and 24 months were purchased from the Animal Center of Chongqing Medical University. All animals were maintained in a controlled facility (20~22°C; 12-hour light/dark cycle) under standard pathogen-free conditions with sterile chow and water *ad libitum*. Animal experiments were performed in accordance with the National Animal Protection and Use Guidelines and were approved by the Animal Ethics Committee of Chongqing Medical University.

### Cell culture

Human aortic endothelial cells (HAECs) were a gift from Nanjing Medical University. HAECs were cultured in Endothelial Cell Medium (ECM) (Cat# 1001, Sciencell, CA, USA), and six to nine generation HAECs with 90% purity were used for experiments.

### Measurement of NO

The NO content in the culture medium was measured using a NO Detection kit and modified Griess reaction method (Beyotime, Shanghai, China) according to the manufacturer’s instructions. Briefly, 50 μL/well standard or sample were added to a 96-well plate followed by Griess Reagent I and Griess Reagent II. The absorbance was measured at 540 nm with a Multiskan Spectrum Microplate Spectrophotometer (Thermo Fisher Scientific, USA).

### SA-β-gal staining

Endothelial cell senescence was identified using a SA-β-gal staining kit (Beyotime, Shanghai, China) following the manufacturer's instructions. Briefly, cells were rinsed once with PBS solution, then fixed in β-galactosidase staining fixative for 15 min. After the fixative was removed, samples were rinsed three times with PBS, then incubated in staining solution overnight at 37? without CO_2_. The staining solution was removed the following day, and cells were immersed in PBS and visualized by phase contrast microscopy (Leica Camera, Germany). ImageJ 1.50i software was used for quantification.

### Western blot analysis

Proteins expression was detected by western blot as described previously [24, 25]. In brief, the aortas or HAECs were lysed with RIPA lysis buffer containing protease and phosphatase inhibitors at 4 ? for 1 h to obtain the proteins. The Bradford method was used to determine the protein concentration, and all samples were diluted to the appropriate concentration for analysis. SDS-PAGE protein loading buffer was added to the protein and heated in a 100 ? water bath for 10 min for the sample preparation. Samples were separated by SDS-PAGE, transferred to PVDF membranes, blocked for 1 h in skimmed milk in TBST, and incubated with the appropriate primary antibodies. The dilution and source of each antibody were as follows: anti -eNOS (1:1,000, rabbit), -p53 (1:1,000, mouse), -p21 (1:500, mouse), -p16 (1:500, mouse), -cGAS (Cat# ab252416, Abcam) (1:1,000, rabbit), -cGAS (Cat# sc-515777, Santa Cruz Biotechnology) (1:1,000, mouse), -p-STING (1:1,000, rabbit), -STING (1:1,000, rabbit), -p-IRF3 (1:1,000, rabbit), -IRF3 (1:1,000, rabbit), and -β-actin (1:2,000, rabbit). Afterward, the membranes were incubated with secondary antibodies conjugated with HRP (goat anti-mouse, 1:10,000; goat anti-rabbit, 1:10,000), and protein bands were visualized by chemiluminescence.

### Vascular reactivity experiments

Vascular vasodilation function was measured as described previously [26, 27]. In brief, aortic rings were mounted and isometric tension recordings were performed using a DMT620 system (Denmark). The rings were placed under a resting tension of 4 mN in a chamber containing warmed (37?), aerated (95% O_2_, 5% CO_2_) physiological saline solution (NaCl 119 mmol/L, KCl 4.7 mmol/L, NaHCO_3_ 25 mmol/L, CaCl_2_ 2.5 mmol/L, KH_2_PO_4_ 1.2 mmol/L, MgSO_4_ 1.2 mmol/L, and glucose 5.5 mmol/L). The integrity of the vascular endothelium was determined by 10^-5^ M acetylcholine (ACh)-induced relaxation in vessels contracted with 10^-7^ M norepinephrine. Concentration-response curves for ACh and sodium nitroprusside (SNP) were performed (10^-9^ to 10^-5^ M). The EC50 and Emax were determined by nonlinear regression analysis using GraphPad V8.0 software (USA). Sensitivity was expressed as pD_2_ = log-EC50.

### Real-time polymerase chain reaction (RT-PCR)

Total RNA was extracted using RNAiso Plus (9109, Takara, Japan). The cDNA synthesis was performed with 2 μg RNA using PrimeScript™ RT reagent kit with gDNA Eraser (Cat# RR047A, Takara, Japan). PCR was carried out using TB Green®Premix Ex Taq™ (Cat# RR820A, Takara, Japan). The following thermal cycling conditions were used: 50? for 2 min and 95? for 10 min, followed by 40 cycles of 95? for 15 s, 60? for 60 s. The following primer sequences were used: IFN-β (sense 5'-ACGCCGCATTGACCATCTAT-3'; antisense 5'-TGGC CTTCAGGTAATGCAGA-3'); Ifit1 (sense 5'-CCTCCTT GGGTTCGTCTACA-3'; antisense 5'-GTTCTCAAAGT CAGCAGCCA-3'); Ifit2 (sense 5'-GACAAGGCCATC CACCACTT-3'; antisense 5'-TCCAGACTCCAAACC CCTCT-3'); Ifit3 (sense 5'-CCCTTCAGGCATAGGC AGTA-3'; antisense 5'-CCTATCGTCCTACCCGTCAC-3'); MCP-1 (sense 5'-GATCTCAGTGCAGAGGCTCG-3'; antisense 5'-TTTGCTTGTCCAGGTGGTCC-3'); TNF-α (sense 5'-GCTGCACTTTGGAGTGATCG-3'; antisense 5'-TCACTCGGGGTTCGAGAAGA-3'); IL-1β (sense 5'-CTTCTGGGAAACTCACGGCA-3'; antisense 5'-AGCACACCCAGTAGTCTTGC-3'); IL-6 (sense 5'-ACCCCCAGGAGAAGATTCCA-3'; antisense 5'-GAT GCCGTCGAGGATGTACC-3'); and β-actin (sense 5'-CCTTCCTGGGCATGGAGTC-3'; antisense 5'-TGATC TTCATTGTGCTGGGTG-3'). Each assay was performed in triplicate and each experiment was repeated 6 times. Data were analyzed by the 2^-ΔΔCt^ method.

### The siRNA transfection

The function of cGAS and STING in D-GAL-induced senescent HAECs was examined by knocking-down cGAS and STING expression using siRNA transfection. All siRNAs were designed using BLOCKiT™ RNAi Designer (Thermo Fisher Scientific, USA) and synthesized by GenePharma Inc. (China).

**Figure 1.**
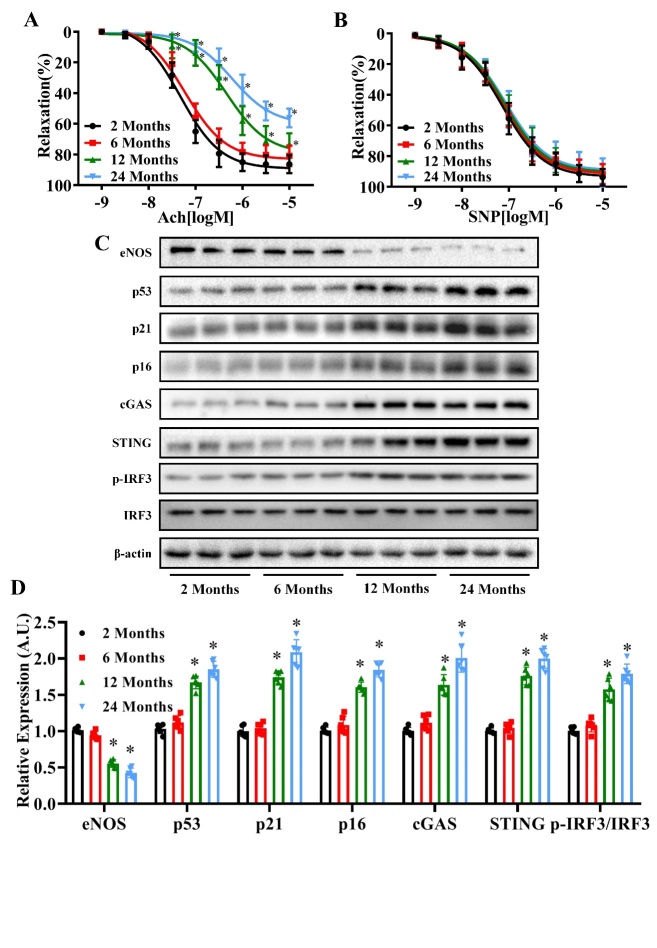
Age-dependent changes in vasodilation function, and eNOS, p53, p21 p16 cGAS, STING and p-IRF3/IRF3 expression levels in mice. Acetylcholine (ACh)-induced relaxation (A) and sodium nitroprusside (SNP)-induced relaxation (B) of mouse aortas were measured in the 2 Months, 6 Months, 12 Months and 24 Months age groups. (C) The protein levels of eNOS, p53, p21, p16, cGAS, STING, p-IRF3, IRF3 and β-actin were measured in the aortas of mice in each age group by western blot analysis. β-actin was used as the housekeeper protein for normalization. Quantification of protein levels is shown in (D). Data were analyzed by (A and B) two-way ANOVA or (D) one way ANOVA plus Bonferroni post hoc test. All data shown are mean±SD. AU indicates arbitrary units. Relative expression is the fold changes relative to the 2 Months group. n=6, ^*^P<0.05 compared with the 2 Months group.

The following siRNA target sequences were used: sicGAS (sense 5'-GGCUAUCCUUCUCUCACAUTT-3'; antisense 5'-AUGUGAGAGAAGGAUAGCCTT-3'); and siSTING: (sense 5'-CAGUGGAUCUAAAUCUCAA ACUGAU-3'; antisense 5'-AUCAGUUUGAGAUUUA GAUCCACU G-3'). Cells were transfected with siRNA using Lipofectamine™ 3000 (Cat# L3000015, Thermo Fisher Scientific, USA) according to the manufacturer’s instructions. In brief, the siRNA transfection was performed when the cell density reached 70%. Lipofectamine™ 3000 and siRNAs were diluted with Opti-MEM™ medium (Cat# 31985070, Gibco, Thermo Fisher Scientifc, USA) in tube A and B, respectively. Next, the liquids in tube A and B were mixed in equal proportions, and the mixture was incubated at room temperature for 15 min. Afterward, the mixture was added into the cell culture medium to initiate transfection and the medium was replaced with the fresh 6 h later.

### Immunohistochemistry

The sections were deparaffinized in xylene, rehydrated in ethanol and subsequently heated in a microwave in sodium citrate to retrieve antigens. 3% peroxide in methanol was used to inhibit the activity of endogenous peroxidases. Then, the sections were block in PBS containing 10% goat serum. Afterward, the sections were incubated with primary antibodies of cGAS (Cat# sc-515802, Santa Cruz Biotechnology), STING, p-IRF3 and CD31 at 4 ? overnight followed by incubation with the corresponding secondary antibodies conjugated with HRP at room temperature for 1 h. To visualize the immunostaining, the sections were incubated in diaminobenzidine followed by a counterstain in hematoxylin. Negative control experiments were done by omitting the primary antibodies. The images were captured by a light microscopy (Leica Camera, Germany). ImageJ 1.50i software was used to quantify the positive staining rate of each sample.

### Immunofluorescence Staining

The immunofluorescence staining was detected using a Tyramide Signal Amplification (TSA) Dual Fluorescent staining Kit (Cat# G1235-100T, Servicebio, Hubei, China) following the manufacturer's instructions. Briefly, the human aorta sections were deparaffinized in xylene, rehydrated in ethanol and subsequently heated in a microwave in sodium citrate to retrieve antigens. 3% peroxide in methanol was used to inhibit the activity of endogenous peroxidases. Then, the sections were block in PBS containing 10% goat serum. Afterward, the sections were incubated with primary antibody of CD31 (Cat# AF20112, AiFang biological) at 4 ? overnight followed by incubation with HRP-conjugated goat anti-mouse secondary antibody for 1 h. The sections were rinsed by PBS for 3 times and then incubated with TSA-FITC staining solution (1 mL Tyramide diluent, 10 μL 0.3% H2O2, 2 μL FITC-Tyramide) for 10 min in a light-free box. Next, the sections were heated in a microwave in sodium citrate followed by incubation with primary antibodies of cGAS, STING, and p-IRF3 at 4 ? overnight. Corresponding HRP-conjugated secondary antibodies were applied to incubate the sections for 1 h, and then TSA-CY3 staining solution (1 mL Tyramide diluent, 10 μL 0.3% H2O2, 2 μL CY3-Tyramide) was used to incubate the sections for 10 min in a light-free box. The nuclei were stained with DAPI, and images were captured by a fluorescence microscope (NIKON, Japan). For immunofluorescence staining of mice aorta, the sections were incubated with primary antibody of p-STING at 4 ? overnight followed by incubation with a FITC-conjugated goat anti-rabbit secondary antibody for 1 h. DAPI was used to stain the nuclei subsequently without incubating another primary antibody. The images of mice aorta sections were captured by a fluorescence microscope (Leica Camera, Germany). ImageJ 1.50i software was used to quantify the mean fluorescence of each sample.

### Statistical analysis

GraphPad V8.0 software was used for statistical analysis. All data were expressed as means ± SD, unless otherwise stated. Shapiro-Wilk normality test was performed to assess the normality of the distribution of data. Due to the small sample sizes, we acknowledge the assessment of normality may not reliable. To calculate the comparisons between 2 groups, Unpaired 2-tailed Student t tests or Mann-Whitney U tests were performed for normally or nonnormally distributed data, respectively. To calculate the comparisons between multiple groups (≥3 groups), one-way analysis of variance (ANOVA) or two-way ANOVA followed by Bonferroni post hoc test was performed for normally distributed data, and the kruskal-Wallis test followed by the Dunn post hoc test was performed for nonnormally distributed data. Number of replicates and other statistical details were reported in the figure legends. Relative expression is relative to the 2 Months group *in vivo* experiments and Control group *in vitro* experiments. *P* < 0.05 was considered statistically significant.

**Figure 2.**
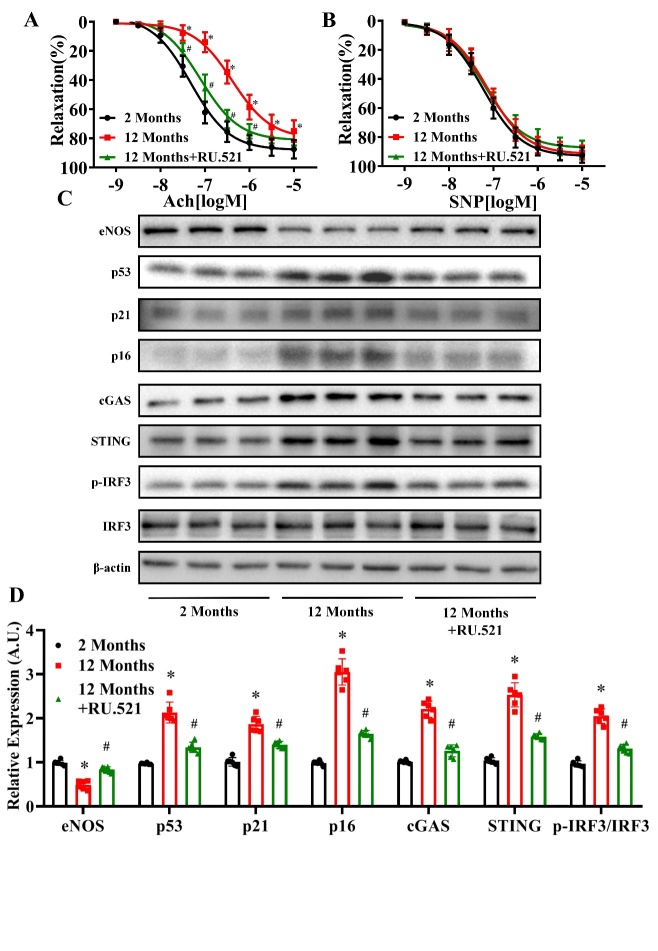
Effects of RU.521 on vasodilation function and eNOS, p53, p21 p16 cGAS, STING and p-IRF3/IRF3 expression levels in senescent mice. Acetylcholine (ACh)-induced relaxation (A) and sodium nitroprusside (SNP)-induced relaxation (B) of mouse aortas were measured in the 2 Months, 12 Months, and 12 Months+RU.521 groups. (C) The protein levels of eNOS, p53, p21, p16, cGAS, STING, IRF3, p-IRF3 and β-actin were measured in the aortas of mice in each group by western blot analysis. The β-actin was used as the housekeeper protein for normalization. Quantification of protein levels is shown in (D). Data were analyzed by (A and B) two-way ANOVA or (D) one way ANOVA plus Bonferroni post hoc test. All data shown are mean±SD. AU indicates arbitrary units. Relative expression is the fold changes relative to the 2 Months group. n=6, ^*^P<0.05 compared with the 2 Months group, ^#^*P*<0.05 compared with the 12 Months group.

**Figure 3.**
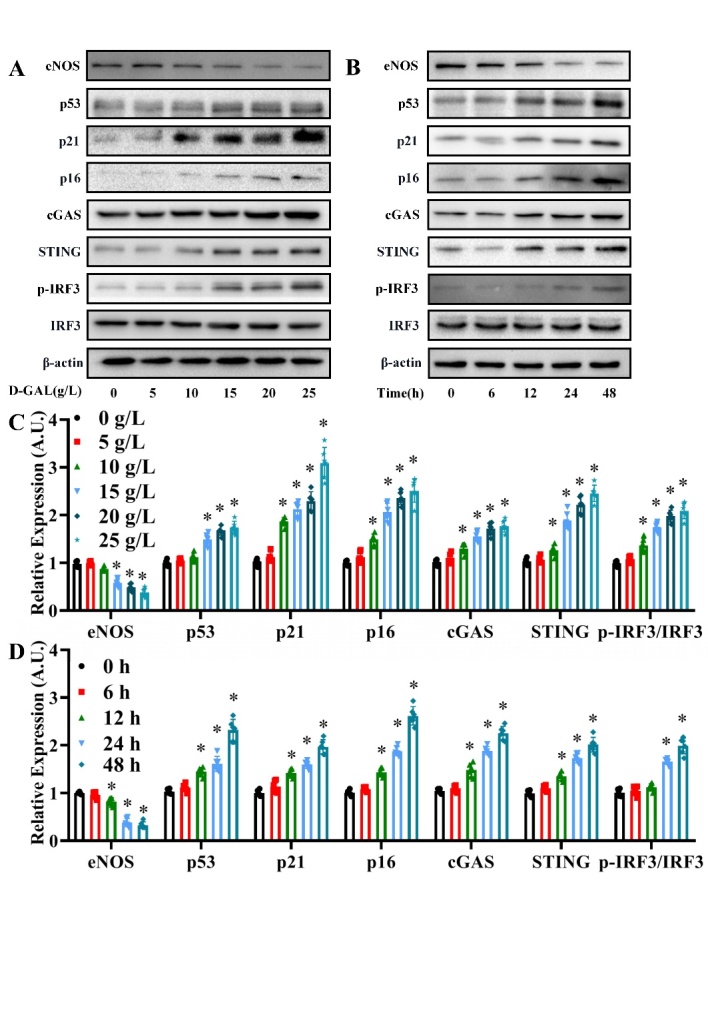
Changes in eNOS, p53, p21, p16, cGAS, STING and p-IRF3/IRF3 expression levels in D-GAL-treated HAECs. (A) The eNOS, p53, p21, p16, cGAS, STING, IRF3, p-IRF3 and β-actin protein expression levels were measured in HAECs treated with 0, 5, 10, 15, 20 and 25 g/L D-GAL for 24 h by western blot analysis. The β-actin was used as the housekeeper protein for normalization. Quantification of the protein levels is shown in (C). Data analyzed by one way ANOVA plus Bonferroni post hoc test. All data shown are mean±SD. AU indicates arbitrary units. Relative expression is the fold changes relative to the 0 g/L group. n=6, ^*^P<0.05 compared with the 0 g/L group. A concentration of 20 g/L was chosen for use in subsequent experiments. (B) The eNOS, p53, p21, p16, cGAS, STING, IRF3, p-IRF3 and β-actin protein expression levels were measured in HAECs at 0, 6, 12, 24 and 48 h after treatment with 20 g/L D-GAL by western blot analysis. The β-actin was used as the housekeeper protein for normalization. Quantification of the protein levels is shown in (D). Data were analyzed by one way ANOVA plus Bonferroni post hoc test. All data shown are mean±SD. AU indicates arbitrary units. Relative expression is the fold changes relative to the 0 h group. n=6, ^*^P<0.05 compared with the 0 h group. A time point of 48 h was chosen for use in subsequent experiments

## RESULTS

### Age-associated changes in vasodilation function and eNOS, p53, p21 p16 cGAS, STING and p-IRF3/IRF3 expression levels in mice

Our aim was to investigate the effects of aging on vasodilation function, senescence markers and the cGAS-STING pathway using mice of different ages. As shown in Fig. 1A, the endothelium-dependent (ACh-induced) vasodilation function declined with age (pD_2_: 2 months: 7.323, 6 months: 7.225, 12 months: 6.338, 24 months: 6.222) (*P*<0.05, n=6), whereas no differences were observed in the function of the endothelium-independent vasodilation (Fig. 1B) (*P*>0.05, n=6). In addition, western blot analysis revealed an age-dependent decrease in eNOS expression, and age-dependent increase in p53, p21, p16, cGAS, STING and p-IRF3/IRF3 protein expression levels (Fig. 1C and D) (*P*<0.05, n=6).

### Effects of RU.521 on vasodilation function and eNOS, p53, p21 p16 cGAS, STING and p-IRF3/IRF3 expression levels in senescent mice

To investigate the role of cGAS in aging-related endothelium-dependent vasodilation dysfunction, a selective cGAS inhibitor, RU.521 (5 mg/kg/day), was injected into 6-month-old mice intraperitoneally for 6 months. We found that inhibition of cGAS significantly prevented the decline in vascular endothelium-dependent vasodilation function compared to the aging group (pD_2_: 2 Months: 7.316, 12 Months: 6.384, 12 Months+RU.521: 7.101) (Fig. 2A) (*P*<0.05, n=6). In contrast, no difference in the function of endothelium-independent vasodilation was observed between the treatment groups (Fig. 2B) (*P*>0.05, n=6). In addition, we found that administration of RU.521 reversed the decrease in eNOS expression and increase in p53, p21, p16, cGAS, STING, and p-IRF3/IRF3 expression levels observed in untreated 12-month-old mice (Fig. 2C and D) (*P*<0.05, n=6). Of note, STING phosphorylation at Ser366 is required for IRF3 binding and activation in the DNA-sensing pathway [28]. As shown in Supplemtary Fig. 1A and B, immuno-fluorescence staining showed that the level of p-STING increased in aortas of 12-month-old mice, which could be reversed by RU.521 administration.

**Figure 4.**
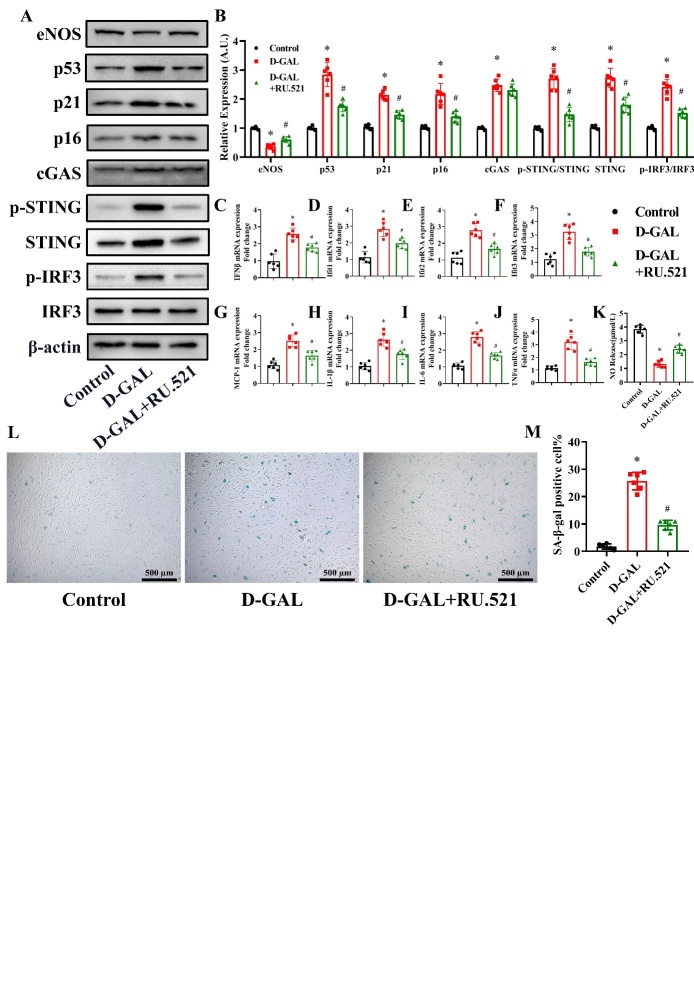
Effects of RU.521 on eNOS, cell senescence, inflammatory cytokines and NO production in D-GAL-treated HAECs. 3μM RU.521 was used to treat the HAECs. eNOS, p53, p21, p16, cGAS, p-STING, STING, IRF3, p-IRF3 and β-actin expression levels in HAECs from the Control, D-GAL and D-GAL+RU.521 groups were examined by western blot analysis (A). The β-actin was used as the housekeeper protein for normalization. Quantification of the protein levels is shown in (B). mRNA expression of IFNβ (C), Ifit1 (D), Ifit2 (E), Ifit3 (F), MCP-1 (G), IL-1β (H), IL-6 (I) and TNF-α (J) in HAECs from the Control, D-GAL and D-GAL+RU.521 groups was determined by PCR. NO release into the culture medium of HAECs from the Control, D-GAL and D-GAL+RU.521 groups was measured (K). Representative photomicrographs and quantitative analysis of SA-β-gal-positive staining in the Control, D-GAL and D-GAL+RU.521 groups (L and M). Data were analyzed by one way ANOVA plus Bonferroni post hoc test. All data shown are mean±SD. AU indicates arbitrary units. Relative expression is the fold changes relative to the Control group. n=6, *P<0.05 compared with the Control group, #*P*<0.05 compared with the D-GAL group.

### Changes in eNOS, p53, p21, p16, cGAS, STING and p-IRF3/IRF3 expression levels in D-GAL-treated HAECs

To examine the effects of senescence on the eNOS and cGAS-STING pathways, HAECs were treated with the senescence inducer, D-GAL. Western blotting analysis revealed a decrease in eNOS expression and concentration-dependent increase in p53, p21, p16, cGAS, STING and p-IRF3/IRF3 expression levels in HAECs treated with D-GAL for 24 h. The most significant effect was observed after treatment with 20 g/L D-GAL (Fig. 3A and C) (*P*<0.05, n=6). Next, HAECs were treated with 20 g/L D-GAL for 0, 6, 12, 24 and 48 h. We found that eNOS expression decreased, while p53, p21, p16, cGAS, STING and p-IRF3/IRF3 protein expression levels increased in a time-dependent manner, with the most significant changes observed after 48 h treatment (Fig. 3B and D) (*P*<0.05, n=6). Based on these findings, a D-GAL concentration of 20 g/L and time point of 48 h were used in subsequent experiments.

**Figure 5.**
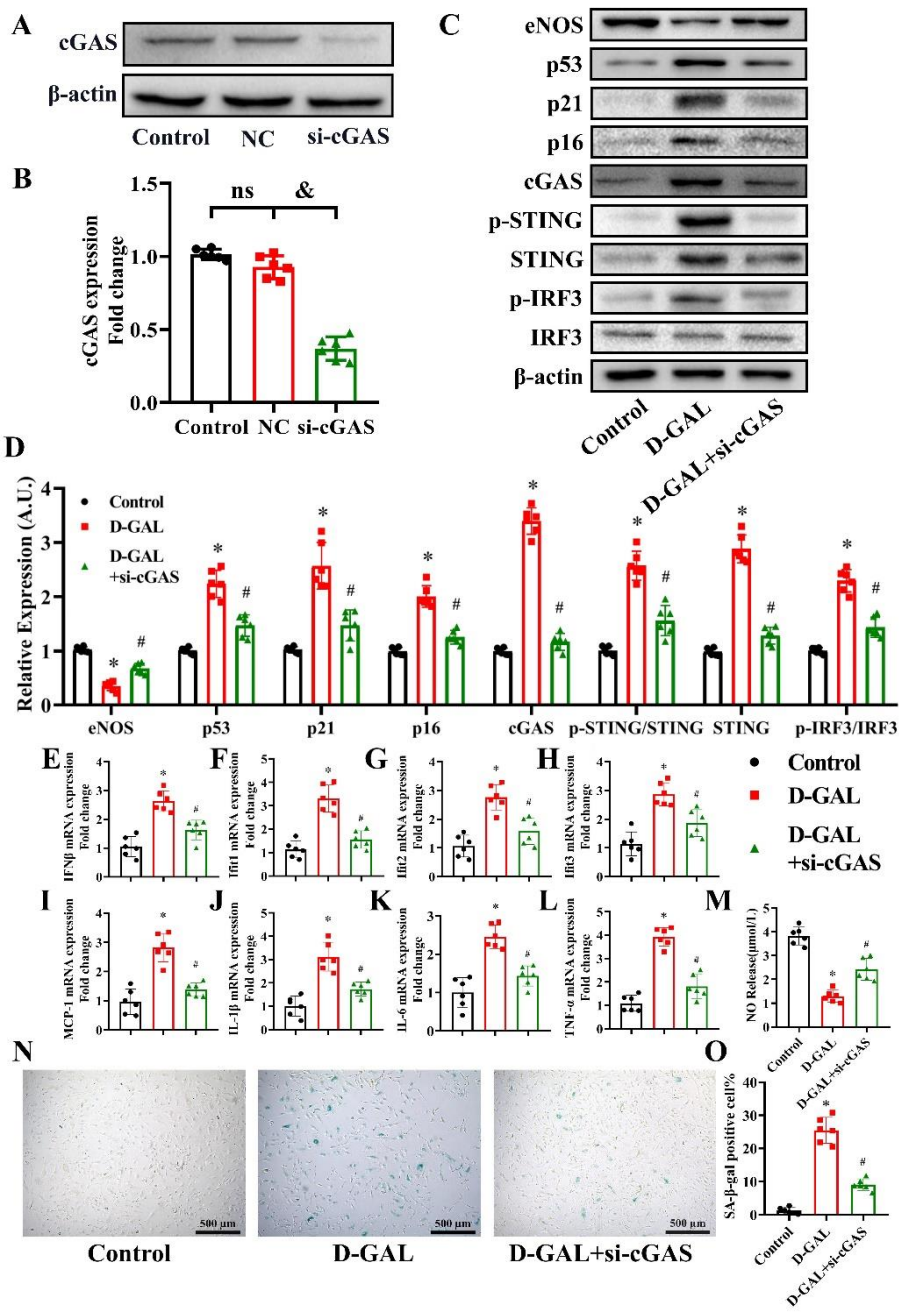
Effects of siRNA-cGAS on eNOS, cell senescence, inflammatory cytokines and NO production in D-GAL-treated HAECs. cGAS expression in the Control, Negative Control (NC) and si-cGAS groups was measured by western blot analysis 48 h after transfection (A). The β-actin was used as the housekeeper protein for normalization. Quantification of the protein levels is shown in (B). Data were analyzed by one way ANOVA plus Bonferroni post hoc test. All data shown are mean±SD. n=6, ^&^*P*<0.05. eNOS, p53, p21, p16, cGAS, p-STING, STING, IRF3, p-IRF3 and β-actin expression levels in HAECs from the Control, D-GAL and D-GAL+si-cGAS groups were examined by western blot analysis (C). The β-actin was used as the housekeeper protein for normalization. Quantification of the protein levels is shown in (D). mRNA expression of IFNβ (E), Ifit1 (F), Ifit2 (G), Ifit3 (H), MCP-1 (I), IL-1β (J), IL-6 (K) and TNF-α (L) in HAECs from the Control, D-GAL and D-GAL+si-cGAS groups was determined by PCR. NO release into the culture medium of HAECs from the Control, D-GAL and D-GAL+si-cGAS groups was measured (M). Representative photomicrographs and quantitative analysis of SA-β-gal-positive staining in the Control, D-GAL and D-GAL+si-cGAS groups (N and O). Data were analyzed by one way ANOVA plus Bonferroni post hoc test. All data shown are mean±SD. AU indicates arbitrary units. Relative expression is the fold changes relative to the Control group. n=6, ^*^P<0.05 compared with the Control group, ^#^*P*<0.05 compared with the D-GAL group.

### Effects of RU.521 on eNOS, cell senescence, inflammatory cytokines and NO production in D-GAL-treated HAECs

We design subsequent experiments to determine the role of cGAS in mediating senescence in HAECs. RU.521, cGAS active inhibitor, at a concentration of 3μM was used to treat the HAECs [29]. As shown in Figure 4A and 4B, Western blot analysis showed that D-GAL induced a decrease in eNOS expression, and increase in p53, p21, p16, p-STING/STING, STING and p-IRF3/IRF3 protein expression levels, which was partly reversed by RU.521 treatment (*P*<0.05, n=6). D-GAL induced an increase in cGAS expression (*P*<0.05, n=6), and RU.521 had little influence on the cGAS expression increased by D-GAL (*P*>0.05, n=6). PCR manifested that the D-GAL treatment led to a significant increase in IFNβ, Ifit1, Ifit2, Ifit3, MCP1, IL-1β, IL-6 and TNF-α mRNA levels, which was partly reversed in RU.521-treated HAECs (Fig. 4C-J) (*P*<0.05, n=6). To examine the effect of D-GAL on NO production by eNOS, we measured the NO content in the cell culture medium of D-GAL treated cells. D-GAL treatment led to a suppression in NO levels, while the levels of NO in the RU.521-pretreated group were higher than those in the D-GAL group (Fig. 4K) (*P*<0.05, n=6). In addition, D-GAL treatment led to a significant increase in the percentage of SA-β-gal-positive cells, which could be reversed by RU.521 treatment (Fig. 4L and M) (*P*<0.05, n=6).

### Effects of si-cGAS on eNOS, cell senescence, inflammatory cytokines and NO production in D-GAL-treated HAECs

We knocked down cGAS by siRNA to further determine the role of cGAS in mediating senescence in HAECs. As shown in Figure 5A and B, si-cGAS significantly silenced cGAS expression in HAECs (*P*<0.05, n=6). Western blot analysis revealed that D-GAL induced a decrease in eNOS expression, and increase in p53, p21, p16, cGAS, p-STING/STING, STING and p-IRF3/IRF3 protein expression levels, which was partly reversed by si-cGAS treatment (Fig. 5C and D) (*P*<0.05, n=6). We also found that D-GAL treatment led to a significant increase in IFNβ, Ifit1, Ifit2, Ifit3, MCP1, IL-1β, IL-6 and TNF-α mRNA levels, which was partly reversed in cGAS-silenced cells (Fig. 5E-L) (*P*<0.05, n=6). To examine the effect of D-GAL on NO production by eNOS, we measured the NO content in the cell culture medium of D-GAL treated cells. D-GAL treatment led to a suppression in NO levels, while the levels of NO in the si-cGAS-pretreated group were higher than those in the D-GAL group (Fig. 5M) (*P*<0.05, n=6). In addition, D-GAL treatment led to a significant increase in the percentage of SA-β-gal-positive cells, which could be reversed by si-cGAS treatment (Fig. 5N and O) (*P*<0.05, n=6).

### Effects of H-151 on eNOS, cell senescence, inflammatory cytokines and NO production in D-GAL-treated HAECs

**Figure 6.**
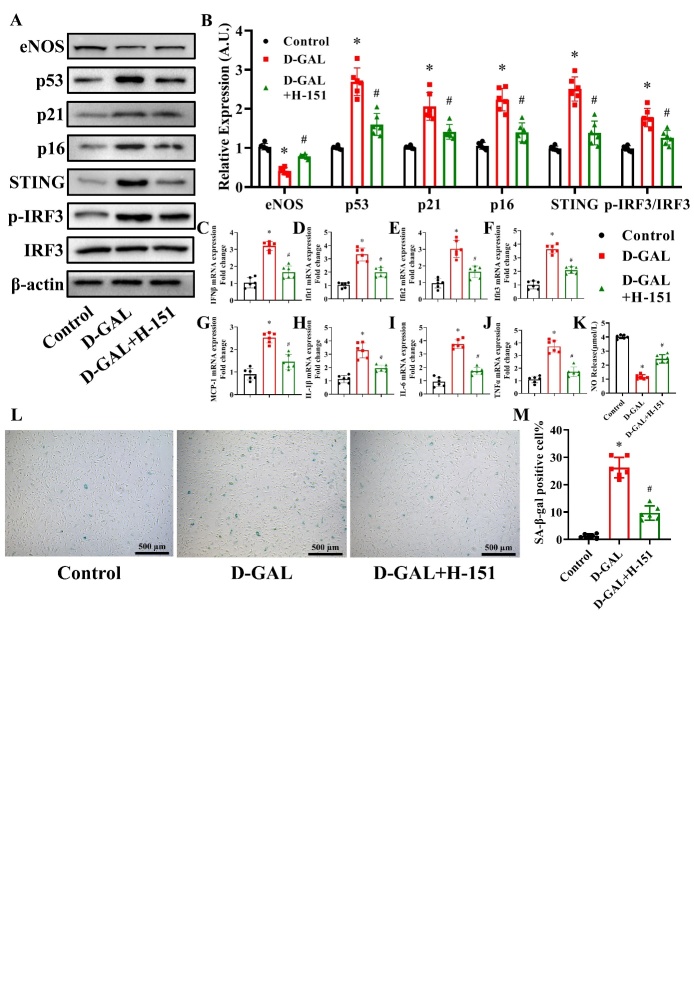
Effects of H-151 on eNOS, cell senescence, inflammatory cytokines and NO production in D-GAL-treated HAECs. H-151 (3 μM) was used to treat HAECs. eNOS, p53, p21, p16, cGAS, STING, IRF3, p-IRF3 and β-actin expression levels in HAECs from the Control, D-GAL and D-GAL+H-151 groups were examined by western blot analysis (A). The β-actin was used as the housekeeper protein for normalization. Quantification of the protein levels is shown in (B). mRNA expression of IFNβ (C), Ifit1 (D), Ifit2 (E), Ifit3 (F), MCP-1 (G), IL-1β (H), IL-6 (I) and TNF-α (J) in HAECs from the Control, D-GAL and D-GAL+H-151 groups was determined by PCR. NO release into the culture medium of HAECs from the Control, D-GAL and D-GAL+H-151 groups was measured (K). Representative photomicrographs and quantitative analysis of SA-β-gal-positive staining in the Control, D-GAL and D-GAL+H-151 groups (L and M). Data were analyzed by one way ANOVA plus Bonferroni post hoc test. All data shown are mean±SD. AU indicates arbitrary units. Relative expression is the fold changes relative to the Control group. n=6, *P<0.05 compared with the Control group, #*P*<0.05 compared with the D-GAL group.

To inhibit the STING expression, 3μM H-151 was used to treat HAECs [30]. As shown in Figure 6A and 6B, D-GAL was found to induce a decrease in eNOS and increase in p53, p21, p16, STING and p-IRF3/IRF3 protein levels, which was partly reversed in H-151-treated cells (*P*<0.05, n=6). D-GAL treatment significantly induced an increase in IFNβ, Ifit1, Ifit2, Ifit3, MCP1, IL-1β, IL-6 and TNF-α mRNA levels, which was partly reversed by H-151 treatment (Fig. 6C-J) (*P*<0.05, n=6). Next, we measured the NO content in the cell culture medium of D-GAL treated cells and found that NO levels were suppressed by D-GAL treatment, while NO levels in the H-151 pretreated group were higher than those in the D-GAL group (Fig. 6K) (*P*<0.05, n=6). Finally, we found that D-GAL treatment significantly increased the percentage of SA-β-gal-positive cells, while H-151 treatment reversed this effect (Fig. 6L and M) (*P*<0.05, n=6).

### Effects of si-STING on eNOS, cell senescence, inflammatory cytokines and NO production in D-GAL-treated HAECs

**Figure 7.**
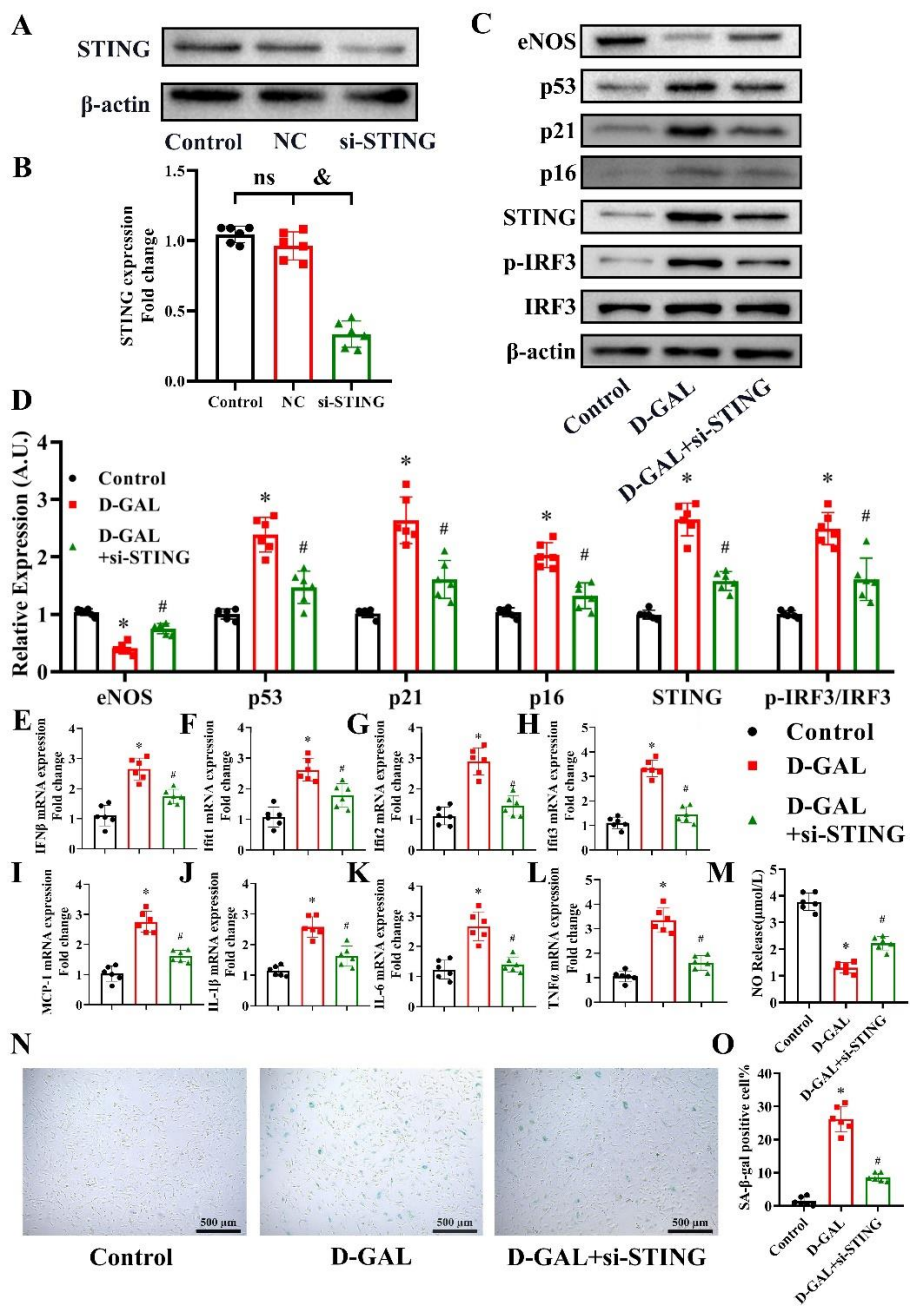
Effects of si-STING on eNOS, cell senescence, inflammatory cytokines and NO production in D-GAL-treated HAECs. STING expression in the Control, Negative Control (NC) and si-STING groups was measured 48 h after transfection (A). The β-actin was used as the housekeeper protein for normalization. Quantification of the protein levels is shown in (B). Data analyzed by one way ANOVA plus Bonferroni post hoc test. All data shown are mean±SD. n=6, ^&^*P*<0.05. eNOS, p53, p21, p16, STING, IRF3, p-IRF3 and β-actin protein expression levels in HAECs from the Control, D-GAL and D-GAL+si-STING groups were examined by western blot analysis (C). The β-actin was used as the housekeeper protein for normalization. Quantification of the protein levels is shown in (D). mRNA expression of IFNβ (E), Ifit1 (F), Ifit2 (G), Ifit3 (H), MCP-1 (I), IL-1β (J), IL-6 (K) and TNF-α (L) in HAECs from the Control, D-GAL and D-GAL+si-STING groups was assessed by PCR. NO release in the medium of the Control, D-GAL and D-GAL+si-STING groups was measured (M). Representative photomicrographs and quantitative analysis of SA-β-gal-positive staining in the Control, D-GAL and D-GAL+si-STING groups (N and O). Data analyzed by one way ANOVA plus Bonferroni post hoc test. All data shown are mean±SD. AU indicates arbitrary units. Relative expression is the fold changes relative to the Control group. n=6, ^*^P<0.05 compared with the Control group, ^#^*P*<0.05 compared with the D-GAL group.

We next knocked-down the expression of STING in HAECs. As shown in Figure 7A and 7B, transfection with si-STING effectively knocked-down STING expression in HAECs (*P*<0.05, n=6). D-GAL was found to induce a decrease in eNOS and increase in p53, p21, p16, STING and p-IRF3/IRF3 protein levels, which was partly reversed in STING-silenced cells (Fig. 7C and D) (*P*<0.05, n=6). D-GAL treatment significantly induced an increase in IFNβ, Ifit1, Ifit2, Ifit3, MCP1, IL-1β, IL-6 and TNF-α mRNA levels, which was partly reversed by si-STING treatment (Fig. 7E-L) (*P*<0.05, n=6). Next, we measured the NO content in the cell culture medium of D-GAL treated cells and found that NO levels were suppressed by D-GAL treatment, while NO levels in the si-STING pretreated group were higher than those in the D-GAL group (Fig. 7M) (*P*<0.05, n=6). Finally, we found that D-GAL treatment significantly increased the percentage of SA-β-gal-positive cells, while si-STING treatment reversed this effect (Fig. 7N and O) (*P*<0.05, n=6).

**Figure 8.**
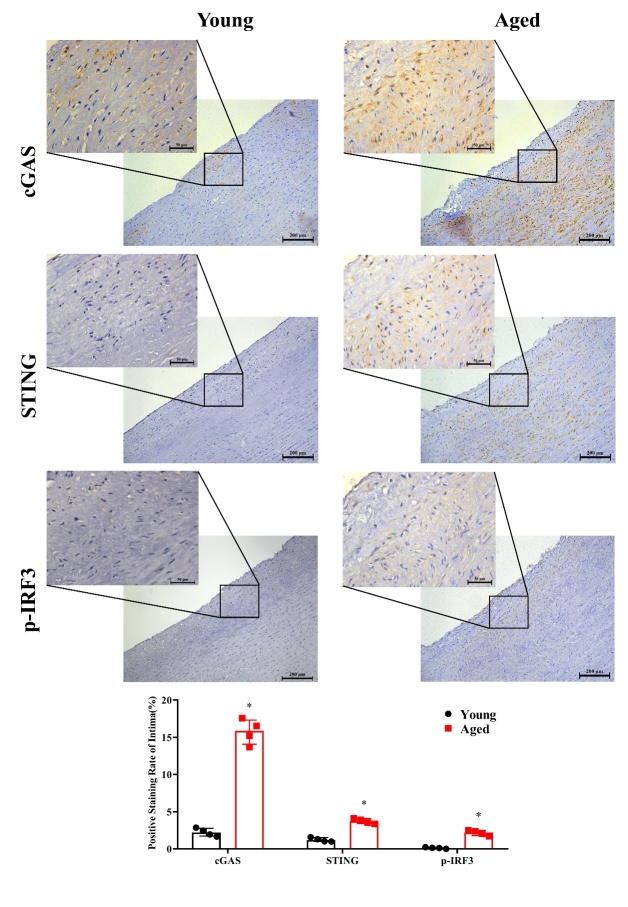
Comparison of cGAS, STING and p-IRF3 levels in young and aged human aortic intima tissue by immunohistochemical staining. Representative photomicrographs and quantitative analyses of immunohisto-chemical staining for cGAS, STING and p-IRF3 in young and aged human aortic intima tissue. Data were analyzed by Mann-Whitney U test. All data shown are median±interquartile range. n=4, ^*^P<0.05 compared with the Young group.

**Figure 9.**
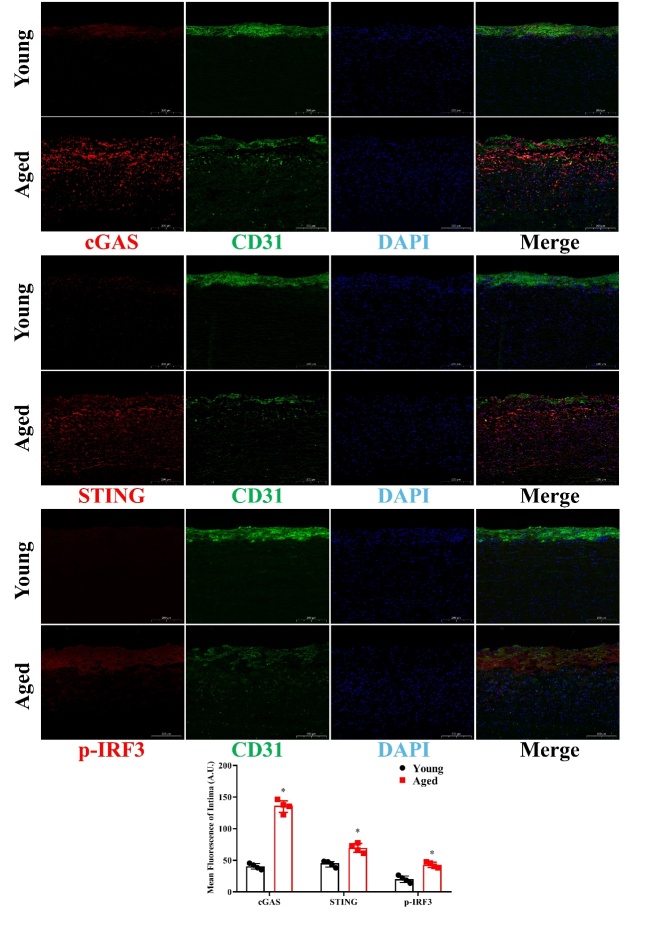
Comparison of cGAS, STING and p-IRF3 levels in young and aged human aortic intima tissue by immunofluorescence staining. Representative photomicrographs and quantitative analyses of immunofluorescence staining for cGAS, STING and p-IRF3 in young and aged human aortic intima tissue. Data were analyzed by Mann-Whitney U test. All data shown are median±interquartile range. AU indicates arbitrary units. n=4, *P<0.05 compared with the Young group.

### Comparison of cGAS, STING and p-IRF3 levels in young and aged human aortic intima tissue by immunohistochemical staining

Immunohistochemical staining was carried out in young and aged human aortic intima tissue to determine age-related differences in cGAS, STING and p-IRF3 in expression levels. We found that cGAS, STING and p-IRF3 levels were significantly higher in aged human aortic intima tissue than young aortic intima tissue (Fig. 8) (*P *<0.05, n=4).

### Comparison of cGAS, STING and p-IRF3 levels in young and aged human aortic intima tissue by immunofluorescence staining

To locate the intima accurately, double immuno-fluorescence staining was carried out, and age-related differences in cGAS, STING and p-IRF3 in expression levels were measured. Endothelial cells were labeled by CD31 and presented as green fluorescence. Red fluorescence represented cGAS, STING, or p-IRF3. We found that cGAS, STING and p-IRF3 levels were significantly higher in aged human aortic intima tissue than young aortic intima tissue (Fig. 9) (*P*<0.05, n=4).

## Discussion

Aging-related cardiovascular diseases, which are prevalent in the elderly population and reduce the quality of life and survival of the old, are becoming an increasingly serious threat to humans due to a rise in the aging population worldwide over the past few decades [31-33]. Endothelial dysfunction, resulting in decline of vasodilation, often occurs with aging and has been reported to be a major cause of cardiovascular disease[34, 35]. Multiple studies have demonstrated that therapies that improve endothelial function and supplement NO production have been beneficial for outcomes of cardiovascular diseases [36, 37]. As eNOS is the key enzyme in producing NO and regulating endothelial function, the expression of eNOS in the vascular system has been directly related to vasodilation function[38]. Here, we found a significant increase in the expression of the senescent markers, p53, p21 and p16, while eNOS expression and endothelium-dependent vasodilation function declined in the aorta in an age-dependent manner. Our findings suggested that aging led to endothelial-dependent vasodilation dysfunction, which is closely associated with the down-regulation of eNOS expression. Thus, determining the mechanism that mediates age-related down-regulation of eNOS expression in blood vessels would be beneficial for improving the vascular function in elderly people.

The process of aging is accompanied by chronic low grade sterile inflammation, which is not caused by a bacterial infection, but by immune dysfunction [13]. The expression of pro-inflammatory cytokines, such as TNF-α, IL-1β and IL-6, has been found to be up-regulated in the large elastic arteries of old mice and humans, and may therefore be associated with age-related cardiovascular disease [39, 40]. Although some studies have shown that activation of nuclear factor-κB (NF-κB) was involved in the up-regulation of pro-inflammatory cytokines in senescent tissue [41], further studies are required to identify other factors that may be involved in this process.

The cGAS-STING is a newly discovered innate immune pathway, which senses and is activated by DNA fragments in the cytoplasm. Activated cGAS can induce downstream STING activation to trigger entry of IRF3 into the nucleus, resulting in the secretion of interferon (IFN). Evidence suggests that mitochondrial dysfunction associated with aging results in the release of mitochondrial DNA (mtDNA) into the cytoplasm [23]. Meanwhile, DNA fragments from nucleic double-strand breaks accumulate in the cytoplasm of senescent cells [42]. Therefore, we hypothesized that the cGAS-STING pathway played a role in the process of aging-related vasodilation dysfunction. We found that cGAS, STING and p-IRF3/IRF3 expression levels were elevated in an age-dependent manner. In particular, because the most significant differences in protein expression were observed between 6- and 12-month-old mice, we used 6-month-old mice in subsequent experiments.

To clarify the relationship between activation of the cGAS-STING pathway and endothelium-dependent vasodilation dysfunction, we treated 6-month-old mice with a specific cGAS inhibitor, RU.521, for 6 months. We found that inhibition of cGAS reversed the decrease in eNOS expression and endothelium-dependent vasodilation dysfunction, as well as the increase in phosphorylation levels of STING and IRF3 and expression of p53, p21, p16, and STING in the aortas of 12-month-old mice. These findings indicated that activation of the cGAS-STING pathway was responsible for the decline in aortic endothelium-dependent relaxation function in aging mice.

The role of the cGAS-STING pathway in mediating senescence in human cells was verified *in vitro*. HAECs were treated with D-GAL to induce senescence [43]. We found that D-GAL treatment resulted in up-regulation of several senescent markers, activation of the cGAS-STING pathway and suppression of eNOS expression in a time- and dose-dependent manner. To clarify the role of the cGAS-STING pathway in aging, we next inhibited or silenced cGAS and STING expression in HAECs. We found that inhibitors or siRNA reversed the D-GAL-induced increase in cellular senescence marker expression and inflammation, as well as the decrease in eNOS expression and NO production. In order to further determine whether the cGAS-STING pathway was involved in the aging process of human tissues, we performed immunohistochemical staining and immunofluorescence staining on human aorta sections and found that the cGAS-STING pathway was activated in elderly patients. It is worth noting that cGAS and STING expression increased in both the intima and middle membrane of the aortas in the aging group. Interestingly, an increase in p-IRF3 levels, the downstream target of cGAS and STING, was observed predominantly in the intima, which is consistent with our hypothesis that aging induces the endothelial dysfunction of blood vessels via the cGAS-STING pathway. Determining the role of increased cGAS and STING expression in the middle membrane should be the focus of future studies.

Of note, *in vitro* experiments showed that RU.521, an cGAS active inhibitor, had no influence on the cGAS expression. However, we found that cGAS levels were lower in the aorta of mice injected with RU.521 for 6 months than in mice in the aging group. This may be due to a reduction in inflammation caused by long-term medication effects, which prevented mitochondrial dysfunction and nucleus DNA damage, reduced mtDNA leakage into the cytoplasm and CCFs generation, and ultimately reduced activation of cGAS. Our findings indicated that a causal cycle may be behind this interesting phenomenon, and future studies will focus on verifying this hypothesis.

Most cardiovascular diseases are associated with vasodilation dysfunction, of which endothelial dysfunction is the most prominent cause [44]. The eNOS is one of the key molecules involved in endothelial function [45]. Therefore, promoting normal functioning of eNOS could be an important strategy to prevent a reduction in vascular relaxation function. Studies have shown that chronic low-grade inflammation and mitochondrial dysfunction appear gradually with age [46]. As blood vessels age, mitochondrial dysfunction causes the release of mtDNA into the cytoplasm and the damage of DNA inside the nucleus leads to an increase in amount of cytoplasmic chromatin fragments (CCFs), which is the basis for activation of the cGAS-STING pathway that induces aseptic inflammation [23, 47, 48]. Indeed, our study verified that the cGAS-STING pathway was activated in the aorta of aging mice, senescent HAECs and aging human aorta tissue sections. Increasing pro-inflammatory factors could lead to a decline in the expression and function of eNOS, a key molecule for vasodilation [1, 27, 49, 50]. These processes eventually lead to a decline in vasodilation function, resulting in increased blood pressure and reduced blood perfusion. In our study, we found that inhibiting activation of the cGAS-STING pathway could significantly prevent the reduction in eNOS, thus protecting the vasodilation function. Our findings provided a novel intervention target for the treatment of age-related cardiovascular diseases.

Interestingly, immunofluorescence staining of human aortas showed that the endothelial cells labeled by CD31 in the aged seemed to be fewer and more diffuse than that in the young, and this phenomenon suggests that loss of endothelial cell characteristics may be another reason resulting in endothelial dysfunction in senescent aortas. Evidence have manifested that endothelial to mesenchymal transition (EndMT) is responsible for down-regulation of CD31 and plays a crucial role in aging-related vascular endothelial dysfunction [51-53]. However, whether cGAS-STING pathway involves in aging-related EndMT remains unknown, and we will try to find out the answer in our future studies. Moreover, we failed to find any changes of CD31 expression among aortas of the mice from 2 Months group, 12 Months group, and 12 Months + RU.521 group (Supplementary Fig. 2), and a recent study on EndMT found a decrease in CD31 expression in aortic endothelium of 18-month-old mice [51]. Therefore, the mice older than 12 months may be more suitable for our subsequent study.

In addition, our study was limited by focused on the silencing of cGAS and STING in cultured HAECs. Thus, further studies using cGAS- and STING-knockout mice are required to examine the role of the cGAS-STING pathway in aging-related vasodilation dysfunction *in vivo*.

In summary, our study demonstrated for the first time that the cGAS-STING pathway was activated in the aorta of aging mice and humans. Activation of this pathway induced aseptic inflammation, decreases eNOS expression and impaired vasodilation function. Our findings suggested that the cGAS-STING pathway may be a novel target for the prevention of age-related cardiovascular disease.

## Supplementary Materials

The Supplementary data can be found online at: www.aginganddisease.org/EN/10.14336/AD.2022.0316.
