# Supplementary material for: Role of the cGAS-STING Pathway in Aging-related Endothelial Dysfunction
Source: Aging Dis. 2022 Dec 1;13(6):1901–18. doi: 10.14336/AD.2022.0316 (PMC9662267; doi:10.14336/AD.2022.0316)
Supplement: Supplementary file 1 — The Supplementary data can be found online at: www.aginganddisease.org/EN/10.14336/AD.2022.0316. [file AD-13-6-1901-s.pdf]

SUPPLEMENTARY DATA

Role of the cGAS-STING Pathway in Aging-related Endothelial Dysfunction

Huilin Yu[1,2], Ke Liao[1,2], Yu Hu[1,2], Dingyi Lv[1,2], Minghao Luo[1,2], Qian Liu[1,2], Longxiang Huang[1*], Suxin Luo[1*]

© 2022. Yu H. et al. Published online at http://www.aginganddisease.org/EN/10.14336/AD.2022.0316

SUPPLEMENTARY DATA

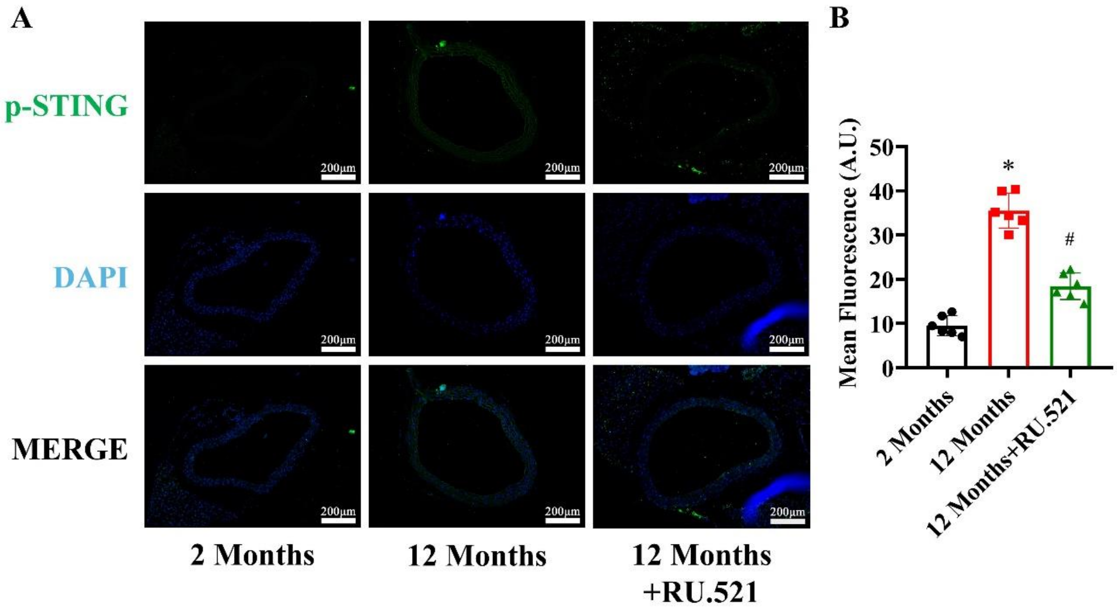

Supplementary Figure 1. Effects of RU.521 on p-STING levels in senescent mice aortas. The selective cGAS inhibitor, RU.521 (5 mg/kg/day), was injected into 6-month-old mice intraperitoneally for 6 months, and immunofluorescence staining was performed on the aortic sections of mice from 2 Months group, 12 Months group, and 12 Months+RU.521 group to measure p-STING levels. Representative photomicrographs (A) and quantitative analyses (B) of immunofluorescence staining for p-STING in aortic sections from each group. Data were analyzed by one way ANOVA plus Bonferroni post hoc test. All data shown are mean ± SD. AU indicates arbitrary units. n=6, **P*<0.05 compared with the 2 Months group, #*P*<0.05 compared with the 12 Months group.

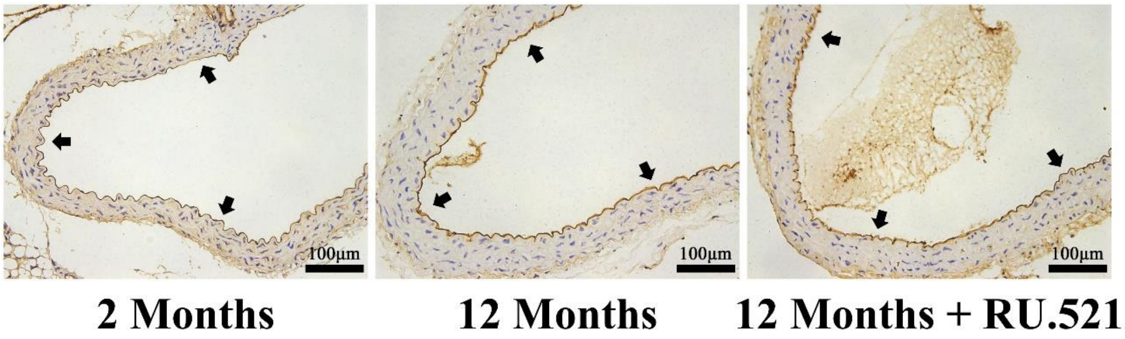

Supplementary Figure 2. Effects of RU.521 on endothelial integrity of aortas in senescent mice. To detect the change of endothelial integrity of mice aortas from each group, CD31 was stained in mice aortas by immunohistochemistry to label endothelial cells. While we failed to find any change of endothelial cell integrity *in vivo* before and after treatment.

© 2022. Yu H. et al. Published online at http://www.aginganddisease.org/EN/10.14336/AD.2022.0316
